# Digital socialization: Insights into interpersonal communication motives for socialization in social networks among undergraduate students

**DOI:** 10.1016/j.heliyon.2024.e39507

**Published:** 2024-10-19

**Authors:** Hailay Tesfay Gebremariam, Paulos Dea, Metasebya Gonta

**Affiliations:** a Department of Ethiopian Languages and Literature (Amharic), College of Social Science and Humanities, Arba Minch University, Arba Minch, Ethiopia; b Department of Psychology, College of Education and Behavioral Studies, Wolaita Sodo University, Wolaita Sodo, Ethiopia

**Keywords:** Digital socialization, Digital technologies, Interpersonal communication motives, Social networks, Socialization behavior, Undergraduate university students

## Abstract

Social interactions and communication between individuals have been completely transformed by digital technology (DT). People from all around the world can now communicate with each other thanks to social media platforms. The main focus of this study is on interpersonal communication motives (ICM) in social networks (SNs), aiming to examine how Ethiopian undergraduate students socialize in relation to digital technologies (DTs). A concurrent design with a mixed approach was utilized, including self-administered questionnaires and focus group discussions (FGD) during the data collection process. The students' ICM were used to gain a deeper understanding of their experiences and perspectives towards digital socialization (DS) in SNs. A total of 312 undergraduates aged 18 to 27 participated in the study, with 202 being male and 110 female. The results of the investigation highlighted the significance of SNs in shaping undergraduate students' ICM. The findings demonstrate the benefits of SNs in facilitating students' socialization process by providing a platform for interaction, communication, and relationship-building. Interestingly, there were no significant variations in ICM in DS using SNs based on gender. By exploring how students socialize within SNs, this study aims to contribute to the existing body of knowledge on the subject. Educational institutions and policymakers could leverage these findings to understand the advantages of incorporating SNs into students' academic experiences, ultimately enhancing their socialization process and overall well-being.

## 1 Introduction

There has been a significant increase in the number of users on social networks (SNs) due to the rapid development of digital technology (DT). With the introduction of socializing behavior through these new technologies, SNs have become a new social arena [[1], [2], [3]]. People can now converse digitally using social media sites in addition to in-person interactions. Nowadays, people use DT for learning, entertainment, staying updated on news, and discussing new trends ([1,[4], [5], [6]]argue that DT has completely transformed the way we perceive communication and engagement. Due to the increasing popularity of SNs [7], individuals can now communicate with others anywhere in the world, regardless of their physical location [8]. This growth has provided consumers with more choices when it comes to digital communication communities. Interpersonal communication (IC) can sometimes lead to misunderstandings as people have different reasons for communicating.

According to Marqués-Sánchez et al. [9], socialization is the process through which an individual develops emotional connections with others and themselves as members of a community. This process occurs through interactions, exchanges, and bonds with other individuals. Due to its significance, Silva et al. [10] suggest that the term has become widely used in literature. Studies by Hardjati and Febrianita [11], Luo and Zhang [12], and Marqués-Sánchez et al. [9] have explored the conceptualization of socialization. Throughout the 2000s, DTs have replaced traditional socializing resources such as friends, family, school, and the workplace [13,14]. The digital generation, also known as the internet generation [15], has been influenced by DTs in terms of their cognitive processes, behaviors, and communications [16]. These generations continue to engage with DTs in various contexts, including relationships, employment, education, and purchasing habits.

Furthermore, DTs have emerged as a powerful tool for socialization that competes with other forms of interaction, significantly influencing people's views and actions, especially among adult college and university students [14]. Members' thoughts, feelings, and actions are impacted by their engagement with DS [17]. This connection is closely tied to both collective efficacy and members' confidence within the community. While DT members may share common interests, they come from diverse age groups, socioeconomic backgrounds, ethnicities, and life stages [9]. Additionally, DTs can be viewed through the social learning theory lens [18], which posits that social interaction plays a vital role in learning participation, defining a community as a social structure. According to Lee et al. [19], Navarro et al. [20], and Wang et al. [18], in terms of communication, individuals are viewed as open communities where members frequently share resources, develop, and exchange knowledge. Knowledge and cognitive shifts are influenced by an individual's involvement in communities of practice [19]. DTs can encompass both communities of interest and communities of practice, as the lines between them are often blurred [14,20].

Despite many studies on ICM focusing on face-to-face interactions [7,13], there has been minimal research on how these incentives impact SNs in mediated contexts. Contrary to previous claims, Gebremariam [22] argued that in-person interactions and the internet are not interchangeable. Additionally, Rubin and Rubin [23] found that when it comes to meeting common interpersonal needs, such as starting a conversation, individuals prefer face-to-face interactions over mediated communication methods. The ICM covered [25] have rarely been studied in relation to mediated communication. Furthermore, incentives relevant towards IC environment have been identified in previous research focusing on social media.

While research on ICM through DTs has focused on the effectiveness and prevalence of outcomes in the digital world [25], not enough attention has been paid to how DS processes and ICM utilization in higher education impact students' technological development [18,26,27]. Research on the influence of ICM on DTs socialization behavior is lacking, especially in Ethiopian contexts. Currently, most DTs focus on skills and prevalence in SNs; however, there is a lack of understanding regarding the relationship between users (e.g., university students) and their ICM in SNs. Additionally, studies show that the majority of college students have poor IC abilities [26]. Higher education students may struggle to use DTs for IC needs if they lack the necessary IC abilities [26,28,29]. Therefore, this study examines the DS for SNs using ICM. The specific research topics are structured as follows.

1. How much do undergraduate university students engage in DTs?
2. How do ICM dimensions impact DS through SNs among undergraduate university students?
3. What effects do gender disparities have on university undergraduate students' ICM of DS?

This study aims to provide a comprehensive understanding of how DTs impact relationships and social interactions among university undergraduate students in SNs. It also explores the effects of DTs on socializing. By gaining insight into these dynamics, the study can inform the development of strategies and interventions to promote positive and meaningful social interactions in the digital age.

## 2 Literature review

### 2.1 Theoretical backgrounds

The Uses and Gratifications perspective has influenced a significant amount of research on DS (See Refs. [30,31]. According to Rubin et al. [32], ICM have recently proposed a connection between social media and in-person encounters as a way to address basic human needs. The original principles that defined the Uses and Gratifications view were identified as: (a) social and psychological origins of (b) human needs, (c) expectations of (d) mass media or other sources, (e) distinct patterns of media exposure or other activities, (f) fulfillment of needs, and (g) other consequences [31]. Each principle can also explain why individuals use IC as a substitute for other means of meeting their needs and motives. In order to quantify face-to-face and digital communication reasons, Rubin et al. [32] defined six factors: control, relaxation, escape, ease, pleasure, and attachment.

There is a clear distinction between needs and motives, although the uses and gratifications approach requires further conceptual development. Human needs for financial support serve as the basis for motives, which are expressed desires for pleasure in specific situations. According to Downs and Javidi [30], individuals may utilize communication channels to fulfill their need for belonging, which is one of the reasons they use these channels to seek companionship. In order to achieve satisfaction, one must first have expectations, or the belief that the action will fulfill the specified need. Distinguishing between pleasures derived from contentment sought has helped clarify this distinction. Satisfaction is based on expectations of enjoyment from consumption, while pleasure is derived from feedback that validates or fulfills a need [14,33].

Three fundamental principles support the media uses and gratifications perspective: media usage is connected to fulfilling needs, media is not the sole source of need satisfaction (think back to the days before social networking), and the audience is purposeful and engaged. Building on the first principle, it is crucial to view the audience as active participants in selecting and using SNs [34]. People intentionally choose items that will bring them the most enjoyment, whether it is a social networking connection. The third assumption is that individuals seek out multiple sources to meet their needs. Various activities can serve the same purpose, and interacting with others can also fulfill needs. In fact, using SNs is often not preferred over meeting needs through close relationships, such as those with family and friends. Instead, individuals may actively seek out opportunities to connect with loved ones to fulfill their needs. Watching or using other media platforms may serve as a substitute for social networking when it is not possible [33,35].

### 2.2 Digital socialization perspectives

Using digital technologies to investigate how people connect and engage with others in social phenomena is the focus of DS theory. According to its classic definition, socialization is a process impacted by culture, digital civilization, and shared traits among all people [36,37]. All valuable DTs aim to facilitate this process. The objective is for individuals to become proficient in perceiving both offline and online realities and integrating their digital persona with their true selves. Consequently, a digital model of socialization blends with the organic socialization of a personality through life events. Conversely, Barreda-Angeles & Hartmann [38] propose that digital socialization should be viewed as a characteristic of contemporary socialization. Similarly, Caballini et al.'s [39] earlier study verifies that socializing on SNs has organized features comparable to offline face-to-face networks. This highlights the process of social integration and adaptation in the context of digitalization, as well as the use of ICM and SNs to acquire new social experiences [40]. Recently, terms like informational socialization, digital childhood, and Barrera-Angeles & Hartmann [38] have emerged in psychology and educational science.

Nowadays, DS is a significant component of traditional socialization and serves as a supplement, similar to how emerging culture complements offline everyday culture [41]. Empirical research suggests that socialization is a situational process impacted by both controlled and uncontrolled variables. Current data show how the digital environment, including information and internet technology, influences the development of social behavior models in the teenage generation [42]. The main focus of this study is the influence of these technologies on interpersonal interactions, communication styles, and the development of online communities [18]. Groups of people with shared interests or goals who primarily communicate online are referred to as DTs, or simply DS [34,37]. However, limitations of face-to-face interactions in offline communities, such as synchrony, physical closeness, and spatial coherences, are overcome by DTs [36,43,44].

Since DT is widely used and accepted in academic contexts [3,45] social support [46], and society at large [47], research on utilizing DTs for social interaction is becoming increasingly important in the field of information systems. Understanding members' behavior in relation to their digital community is a primary goal of DT behavior study [12,48,49]. This is crucial because the ICM behaviors of DT members significantly impact the organization's development and contribution. To further understand the behavior of DS, several researchers have sought a theoretical framework. Factors such as a sense of affiliation or community, a sense of belonging, and emotional states have been linked to DT behavior [5,17,49,50]. Other research has explored social identity theory, self-presentation, and the trust viewpoint. Like offline communities, DTs can serve as venues for establishing connections [35].

### 2.3 Interpersonal communication motives

Regarding online and virtual communication, DT is a product of modern society, especially the informational and electronic society [44,51]. Some view this form of communication as a parallel yet artificial world, while others see it as unreal. Connecting digital communication to the original meanings and relevance of IC is a key aspect of its nature [12]. Various factors related to basic human needs have been identified as causes of IC [17]. Previous research has identified 18 communication reasons [51,52]. Rubin's [53] study on television viewing motivations revealed nine of these reasons, while the remaining nine were sourced from other studies [23]. Rubin et al. [32] outlined motivations such as alertness, escape, self-learning, convenience, social norms, companionship, relaxation, habit, entertainment, passing the time, social interaction, receiving information, altruism, control, self-esteem, safety, and emotional expression.

The majority of research in ICM focuses on examining respondents representing different age groups or the general population. Since the introduction of the ICM scale [32] and model [54,55], there has been increasing evidence supporting reasons why people engage in conversation. According to the ICM theory, people connect based on six reasons: pleasure, affection, inclusion, escape, relaxation, and control [32]. Pleasure involves communicating for enjoyment and social benefits, while affection is driven by gratitude and concern [35]. Inclusive communication stems from a desire to interact and share with others. Communication for escape is driven by the need to pass time. Relaxation communication serves therapeutic purposes for decompression and unwinding. Control communication asserts authority or compels compliance [20,32]. The typology of motivations established by Rubin et al. [32] has been widely applied in ICM research, with additional studies exploring other reasons for communication [20,53,56,57]. The choice of motivation influences who people talk to, what they talk about, and how they communicate [54]. Pleasure, affection, and inclusion are seen as justifications for communication in personal and intimate relationships [54,58,59]. Individuals who communicate for inclusion, affection, and enjoyment tend to be satisfied with their relationships, happier, healthier, and more socially engaged compared to those with other motivations [23,[60], [61], [62]].

However, human connections are often the driving force behind escapism, leisure, and a sense of control [33,41]. Individuals who seek to communicate for control and escape tend to exhibit traits such as an external locus of control orientation [2,23,30], expressive and negative humor, and a dominant communicator style [20,54]. Additionally, the level of satisfaction derived from relaxation is not significantly impacted by the use of these two motivations [32]. A more friendly and animated communicator style, along with lower levels of loneliness, are associated with communication related to relaxation [30,54].

Within the digital society, IC is crucial. It is a constant element of IC where people share information in various settings such as public spaces, workplaces, schools, and colleges [56,64,65]. Social interaction enhances communicators' knowledge, making it easier to share experiences and gain meaning. For most individuals, eighty to ninety percent of their waking hours are spent in IC [49]. Additionally, people in IC inevitably communicate through social media platforms [4,27,66]. The interpretation of news, advice, requests, and suggestions by recipients is influenced by the content and culture of digital messages. Communication through specific digital channels is common [50,65,67]. Communication in DTs takes various forms: in-person interactions (such as in class or university groups), mass media (like TV and radio), lectures, speeches, and information broadcasts [72]. Factors such as time, place, family dynamics, gender, culture, personal interests, and communication environment all play a role in influencing IC.

### 2.4 The current study

Since the ICM was first introduced, a number of studies have provided further evidence in support of its factor structure [4,68] and its associations with age [3,11], communication apprehension [4,6], degree of loneliness [69], contextual age [8], locus of control [10,70], and general communication [32,70]. ICM is important and vital in universities, almost like a second family for students. Students face several challenges while attending university, and building a sense of trust and belonging in them requires a caring and supporting ICM [20,58,62,68,70]. Through trust, students may grow trust amongst themselves, their peers, and DTs worldwide. When it's done right, open communication among college students cultivates trust. Building trust amongst students is highly dependent on open communication [28,39]. Furthermore, according to Barreda-Ángeles and Hartmann [38] the message is the element that is transmitted from one person to another in the ICM theory. Therefore, the purpose of this work is to understand the motives behind human communication and explore potential sources of fulfillment for those motives in DS. The specific hypotheses are as follows.

H1 A significant number of undergraduate students engage in using DS due to ICM factors.

H2 ICM factors impact the engagement in using DS among undergraduate students.

H3 Gender differences in ICM between males and females have a significant impact on undergraduate IC in using DTs.

## 3 Methodology

### 3.1 Research design

The purpose of this study is to investigate undergraduate students' motives for intercultural communication in the context of study abroad programs. The study utilized a concurrent (convergent or parallel) strategy to achieve its research goals by exploring the research question from multiple perspectives. Mixed methods were employed in the concurrent/convergent design to combine qualitative and quantitative data in a single research study or set of studies, researchers can gain a more comprehensive understanding of a topic compared to using either method alone [73,74]. This approach allows for a richer analysis and interpretation of findings leading to more robust conclusions. Additionally, the mixed method can help to validate the results and enhance the overall credibility of the study by utilizing both quantitative and qualitative researchers can address the limitations of each approach and provide a more well-rounded investigation. To enhance the comprehensiveness of the research and ensure the validity and reliability of the findings, it is beneficial to employ a variety of methodologies in the study of intercultural communication in study abroad programs [45]. Therefore, data was gathered from research participants through an ICM questionnaire using a quantitative approach. Additionally, to obtain detailed insights from a specific group of participants, a qualitative method involving focus group discussions was also utilized.

### 3.2 Participants and context

Convenience sampling was used to recruit the research participants, where individuals were chosen based on their availability and willingness to participate, making it a non-probability sampling technique. Researchers approached students on campus to invite them to participate in the study. The sample consisted of 277 undergraduate students from various colleges at Arba Minch University in Ethiopia to ensure adequate representation of the student body to collect data through questionnaires. Participants were selected from different academic levels and departments to create a diverse sample, including students from both humanities and sciences. The research included participants from various academic years to provide a comprehensive perspective. Among the 277 undergraduate participants 12 students were selected to participate in FGD by constructing into two groups. The FGD was enrolled after the questionnaire collected.

### 3.3 Data collection tools

This study utilized questionnaires based on the ICM and DS scales, and focus group discussions (FGDs), as a data collection methods. The surveys were created to gather quantitative information from undergraduate students regarding their ICM during the DS period. The questionnaire included sociodemographic information, participants' reasons for using IC, and their familiarity with social media platforms.

**Socio-demographic information**: After introducing the general questionnaire format, data on respondents' socio-demographic characteristics such as age, gender, college or university, field of study, university year level, social network usage, experience with technology, preferred device, time spent on Sns, duration of use, frequency of checking social network accounts, and hours spent on Sns were collected (Refer to Table 1).

**Table 1:** Socio-demographic information of respondents (N = 277).

Items with Options	Frequency	Percent	Items with Options	Frequency	Percent
SNs use			Preferred device you use		
Yes	277	100	Desktop computer	9	3.2
No	–	–	Laptop	66	23.8
Gender			Mobile	202	72.9
Male	202	72.9	SNs account actively using		
Female	75	27.1	1-2 accounts	100	36.1
Age			3-4 accounts	102	36.8
Below 20 Years	33	11.9	More than 4 accounts	75	27.1
20–24 Years	195	70.4	Duration of using SNs		
Above 24 Years	49	17.7	Less than 1 year	44	15.9
College/University			1–2 years	124	44.8
Social Science/Humanities	85	30.7	3–4 years	78	28.2
Business and Economics	94	33.9	More than 4 years	7	2.5
Medicine/Health Science	67	24.2	Checking Sns account per day		
Natural Science	23	8.3	on every notification beep	50	18.1
Other	8	2.9	1-2 times per day	149	53.8
Program of Study			3-4 times per day	46	16.6
Regular/Day	184	66.4	5-6 times per day	19	6.9
Extension/Weekend/Night	93	33.6	More than 6 times per day	6	2.2
Years level in University			SNs use hours per day		
1st Year	64	23.1	less than 1 h	98	35.4
2nd year	92	33.2	1–2 h	122	44.0
3rd year	107	38.6	3–4 h	51	18.4
4th year	14	5.1	5–6 h	6	2.2
Experience with technology facilities			More than 6 h	–	–
Beginner	178	64.3			
Average	85	30.7			
Expert	14	5.1			

**Familiarity with social network platforms:** The second segment evaluated undergraduate students' knowledge of various social networking platforms, including voice chat (like Google Hangout), video chat (like Zoom, Skype), web video (like YouTube), social media (like Facebook, WhatsApp), and professional web (like LinkedIn). Respondents could choose from “Always use it (5)" to “Almost never use it (1)" on a Likert scale.

**Interpersonal communication motives**: The study utilized a questionnaire developed by Rubin et al. [32], previously used by other researchers (e.g., Refs. [30,52,75]. This questionnaire consisted of twenty-eight Likert-type items that assessed reasons why individuals engage with others on SNs. These reasons fell under six components: pleasure, attachment, inclusion, escape, relaxation, and control. Sample items include “I communicate with digital communities to have fun” and “I communicate with digital communities to enjoy myself.” Responses were rated on a 5-point Likert scale from 1 (Never) to 5 (Always).

A principal components analysis with Varimax rotation was conducted to explore the underlying structure of the 28 items. Factor extraction was based on minimum communalities of 0.74 and minimum 60/40 loadings per factor. Ultimately, five factors and 24 items were retained, explaining 60.77 % of the total variance. Factor loadings corresponding to the retained items are presented in Table 3. Factor 1, labeled “pleasure” (Eigenvalue = 9.21, Cronbach's alpha = 0.90), comprised seven questions related to seeking enjoyment during online interactions. This component explained about 34.32 % of the total variance. Internal reliability of the ICM scale ranged from 0.72 for inclusion to 0.87 for pleasure [32]. Internal reliabilities for each factor in this study were as follows: pleasure (0.87), affection (0.79), inclusion (0.72), escape (0.76), relaxation (0.83), and control (0.77).

**Table 2:** Descriptive statistics through means and standard deviation for familiarity with SNs (N = 277).

Familiarity with SNs	M	SD	Always	Often	Sometimes	Rarely	Never
Search engine (e.g., Gmail, Google, Ask.com, Yahoo)	3.01	1.19	72 (26 %)	86 (31 %)	53 (19.1 %)	36 (13 %)	1 (0.4 %)
Voice chat (e.g., Google hangout, Viber, teamSpeak)	2.83	1.25	54 (19.5 %)	71 (25.6 %)	76 (27.4 %)	43 (15.5 %)	1 (0.4 %)
Video chat (e.g., Zoom, Skype, Google Meet)	3.35	1.22	68 (24.5 %)	75 (27.1 %)	54 (19.5 %)	18 (6.5 %)	3 (1.2 %)
Web video (e.g., You Tube)	3.31	1.01	94 (33.9 %)	92 (33.2 %)	52 (18.8 %)	7 (2.5 %)	2 (0.8 %)
Social Media (e.g., Facebook, TikTok, Telegram, X)	3.24	1.11	80 (28.9 %)	97 (35 %)	44 (15.9 %)	19 (6.9 %)	5 (2 %)
Professional web (e.g., LinkedIn)	3.24	1.12	78 (28.2 %)	85 (30.7 %)	58 (20.9 %)	15 (5.4 %)	7 (2.8 %)

**Table 3:** Descriptive statistics through means and standard deviation for ICM and DS (N = 277).

Variables		M	SD	Skewness	Kurtosis	Status/Level
Interpersonal communication motives	Pleasure	3.26	0.64	0.188	−0.333	Moderate
	Affection	3.36	0.65	0.147	0.005	Moderate
	Inclusion	3.21	0.68	0.426	−0.052	Moderate
	Escape	3.48	0.69	0.118	−0.633	Moderate
	Relaxation	3.60	0.68	−0.130	−0.345	Moderate
	Control	3.55	0.75	0.022	−0.695	Moderate
Digital Socialization	Socialization	3.41	0.61	0.291	−0.188	Moderate

**Digital Socialization Scale**: Gupta and Bashir [8] developed the scale used in this study, validated by Khan et al. [76]. This scale consisted of six Likert-type questions assessing university students' digital socialization. Sample items included “I prefer using SNs over attending social gatherings.” Responses were rated on a 5-point Likert scale from 1 (Never) to 5 (Always). A principal components analysis with Varimax rotation was conducted to examine the underlying structure of the six items. Following guidelines of at least 50/40 loadings per factor and minimum communalities of 0.67, three items were retained, explaining 51.32 % of the total variance. The internal reliability of the DS scale has been previously shown to be 0.82.

**Focus Groups Discussions**: FGDs involved a select group of participants from each college within the chosen institution, serving as an additional data collection technique. The selected students who participate of were confirmed to be familiar with SNs. Three FGDs were conducted with five members in each group to validate and supplement the quantitative data. FGDs are known to provide rich qualitative data that may not be captured through other methods. Semi-structured questions were prepared based on the specific objectives outlined earlier.

### 3.4 Validity and reliability of instruments

Before the questionnaire was administered, it was piloted to ensure clarity and reliability. This was achieved by utilizing Cronbach's alpha, Confirmatory Factor Analysis (CFA), and Exploratory Factor Analysis (EFA) to assess the scale and items' reliability. Participants in the pilot study were asked for feedback to determine if any changes were necessary to improve the tools for the actual study. To validate that the tools effectively obtain the desired information in line with the study objectives, they were sent to colleagues for feedback and evaluation.

An analysis using principle components analysis with Varimax rotation was conducted to examine the underlying structure of the 28 items. The factor extraction process adhered to minimal communalities of 0.74 and a minimum 60/40 loadings per factor. Ultimately, 24 items and five variables explained 60.77 % of the total variation. Specifically, Factor 1, labeled pleasure (Eigenvalue = 9.21, Cronbach's alpha = 0.87), consists of eight elements representing individuals' motivations for seeking enjoyment in online interactions or digital socializing. This factor explains approximately 34.32 % of the overall variation. Factor 2, labeled affective (Eigenvalue = 6.33, Cronbach's alpha = 0.65), consists of five elements representing individual motivation for sensitivity in DS. This factor explains 25.8 % of the overall variation. Factor 3, labeled escape (Eigenvalue = 8.04, Cronbach's alpha = 0.77), consists of four elements representing individuals' DS use in terms of their break from any control of others in DS, explaining approximately 28.23 % of the overall variation. Factor 4, labeled inclusive (Eigenvalue = 7.46, Cronbach's alpha = 0.68), consists of four elements representing controlling their emotions in the DS, explaining 31.81 % of the overall variation with the other variables. Factor 5, labeled relaxation (Eigenvalue = 8.98, Cronbach's alpha = 0.81), consists of four elements representing the enjoyment in DS. The factor explains approximately 36.23 % of the overall variation. Factor 6, labeled control (Eigenvalue = 9.21, Cronbach's alpha = 0.90), consists of three elements representing the DS use in online networking systems. This factor explains approximately 29.69 % of the overall variation. Factor 7, labeled DS which is the dependent variable of the study (Eigenvalue = 9.87, Cronbach's alpha = 0.85), consists of six elements representing the SNs use of undergraduate students at Arba Minch University. This factor explains approximately 33.35 % of the overall variation.

### 3.5 Methods of data analysis

Both descriptive and inferential statistics were used to analyze the data obtained from the surveys. Descriptive statistics were used to compile demographic data and IC motivations, while inferential statistics, such as correlation analysis, were used to investigate the association between these variables. For analysis, the quantitative information collected through the questionnaire was entered into AMOS 23. The unit of analysis was the mean of the items. A one-sample *t*-test was used to determine if university students' ICM in DTs was significantly above or below the predicted mean values. The ICM dimensions of competency in the DT socialization process were compared using a one-way ANOVA. Additionally, the relationship between university students' ICM in DTs was assessed using the Pearson product-moment correlation. A significance threshold of five percent (α = 0.05) was maintained throughout the study. Furthermore, the effect size test (Cohen's d) was used to determine the degree of variation in the mean scores across all levels.

The socialization process of university students in DTs was explored through ICM using data from FGDs. The qualitative data collected during focus group discussions was coded, categorized, and analyzed using verbal descriptions. Specifically, Gebremariam and Sisay [15] used the thematic analysis of focus group the following six steps: (1) familiarization with the data by reading and rereading it to gain a deep understanding of the content; (2) generating initial codes that are assigned to segments of data based on their content. In this step, initial codes are generated to capture the key ideas and concepts present in the data; (3) searching for themes: Themes are patterns or trends that emerge from the data. In this step, researchers identify and define themes that represent the main ideas or concepts in the data; (4) reviewing themes: Once themes have been identified, researchers review and refine them to ensure they accurately reflect the data; (5) defining and naming themes: In this step, researchers define and name each theme to clearly communicate its meaning and significance; (6) writing the report: The final step involves writing a report that presents the findings of the thematic analysis, including a detailed description of the themes and supporting evidence from the data. To provide a comprehensive understanding of the topic, the findings from both quantitative and qualitative approaches were combined in the discussion section.

### 3.6 Self-selection bias

The self-selection bias in this study is based on cross-sectional data and a self-reported single source, which is theoretically relevant as it focuses on the digital socialization of university undergraduate students. The amount of illicit covariance that is shared is described when researchers conduct their research using self-selection [78]. This occurs as a result of data collection from the same study being done in a consistent manner [79]. According to ICM studies, self-selection reports are defined by digital socialization, and they follow the advice to use perceptual cures both before and after the data gathering procedure. The authors implemented many strategies to address prevalent concerns about potential source bias during the data collection process. Bias in self-selection was minimized by limiting the survey instrument to noncomplex measures with well-established psychometric properties and clearly labeled response choices. Separating predictors and dependents in the questionnaire created a psychological long time, and emphasizing students' voluntary and anonymous participation in the cover letter reduced social desirability. In the end, a number of statistical corrective measures were implemented using the items in the questionnaire and the common factor test via CFA in order to diagnose any common source bias by conducting a single factor. **Ethical Consideration**.

The data in the manuscript is original and accurately depicts findings from human participants. Prior to the data collection began, participants were informed that their involvement was voluntary and that the results would be used for educational research purposes. Written informed consent was obtained from all participants, who were assured that their information would be kept confidential and used exclusively for the study. The study's ethical protocol was reviewed by two senior professors and approved by the Department Graduate Committee (Ref. No.: PSD/251/16) of the Psychology Department at Wolaita Sodo University.

## 4 Data analysis and results

In order to participate in the research, the study's participants were required to give their consent. Initially, a reputable organization sent a letter to the researcher requesting cooperation from selected colleges. Once approval was obtained, the researcher reached out to department heads and selected subjects. These individuals were then briefed on the study's objectives and the type of information that would be required. Additionally, participants were assured that any confidential information provided would be kept private and not shared without their permission. Once participants agreed to take part in the study, a questionnaire was distributed and a focus group discussion was conducted with selected participants.

### 4.1 Respondents’ questionnaire results

The study's findings shed light on the reasons behind information sharing behavior among college students. These findings contributed to the understanding of the factors that influence students' motivation to engage in information sharing with academics and researchers. To achieve this, three research questions were formulated, and self-reported questionnaires were used to collect data from university students. The data was then analyzed using descriptive and inferential statistics, including MANOVA analysis and Pearson correlation.

**Fig. 1:**
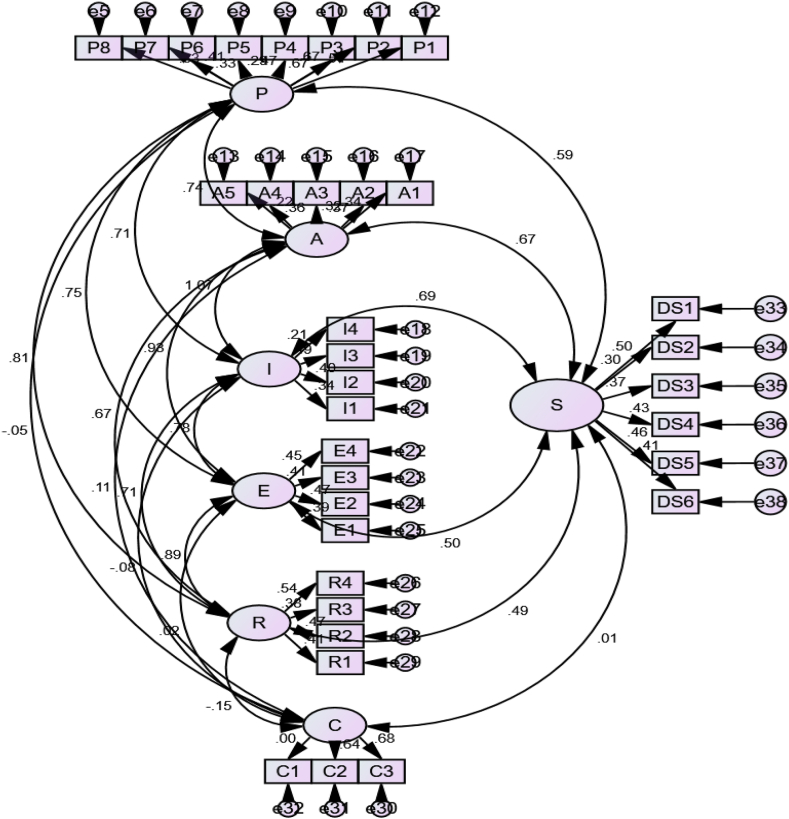
CFA path diagram of study results. Note: P = pleasure, A = affective, E = escape, C = control, R = relaxation, I = inclusive, S = digital socialization.

Fig. 1 shows that the results of the CFA indicate that the model had good fit statistics, including X2/df = 3.03, RMSEA of 0.013, RMR of 0.002, and GFI of 2.325. The recommended values, based on the guidelines of Browne and Cudeck [80], are provided in the brackets (RMSEA <0.05, RMR <0.05, GFI >0.90). All items' standardized factors had loadings above 0.50, indicating good convergent validity. Another evidence of convergent validity is that the maximum shared variance is less than the respective average variances extracted for all variables. The Cronbach's alpha and composite reliability for all variables are above 0.65, demonstrating good reliability [81].

Table 2 presents data on participants' ICM and level of acquaintance with Sns overall. In terms of familiarity, 57 % of participants stated they used search engines frequently (31 %) or always (26 %), compared to 19.1 % occasionally, 13 % infrequently, and 0.4 % never, totaling 32.5 %. Regarding voice chat, 19.5 % stated they always use it, 25.6 % use it frequently, and 43.3 % of respondents reported familiarity with voice chat, below average at 45.1 %. Eleven percent of participants did not choose any option from the never to always list. Video chat was more common (51.6 %), with 24.5 % using it constantly, 27.1 % frequently, and the remaining percentages at 19.5 % sometimes, 6.5 % infrequently, and 1.2 % never. Web video had the highest familiarity rate, with 33.9 % always, 33.2 % frequently, and 22.1 % other responses, totaling 67.1 %. 63.9 % of respondents reported using social media more frequently (28.9 % always, 35 % often), compared to 15.9 % occasionally, 6.9 % infrequently, and 2 % never. A familiarity rate of 58.9 % was found for professional websites, with 28.2 % usually, 30.7 % often, 20.9 % sometimes, 5.4 % infrequently, and 2.8 % never. With the exception of voice chat technology, which was less common, most respondents were familiar with SNs.

The study's factors are presented in Table 3 along with descriptive statistics. The findings include the mean, standard deviations, skewness, and kurtosis for pleasure (M = 3.26; SD = 0.64; Skewness = 0.188; Kurtosis = −0.333), affection (M = 3.36; SD = 0.65; Skewness = 0.147; Kurtosis = 0.005), Inclusion (M = 3.21; SD = 0.68; Skewness = 0.426; Kurtosis = −0.052), escape (M = 3.48; SD = 0.69; Skewness = 0.118; Kurtosis = −0.633), relaxation (M = 3.60; SD = 0.68; Skewness = −0.130; Kurtosis = −0.345), and control (M = 3.55; SD = 0.75; Skewness = 0.022; Kurtosis = −0.695). The descriptive statistics suggest potential significant correlations between the mean values, ranging from 3.21 to 3.60, across all the data and it shows that the university undergraduate students' ICM is moderate to use DS. To assess statistically significant positive correlations between the ICM and DS factors, as displayed in Table 4, inferential statistics were utilized.

**Table 4:** Correlation Matrix for the university students’ ICM and DS patterns.

Variables	1	2	3	4	5	6	7	M	SD
1. Pleasure	1							3.26	0.64
2. Affection	0.420^a^	1						3.36	0.65
3. Inclusion	0.364^a^	0.399^a^	1					3.21	0.68
4. Escape	0.461^a^	0.379^a^	0.358^a^	1				3.48	0.69
5. Relaxation	0.500^a^	0.290^a^	0.329^a^	0.431^a^	1			3.60	0.68
6. Control	0.082	0.189^a^	0.104	0.162^a^	0.088	1		3.55	0.75
7. Socialization	0.384^a^	0.311^a^	0.309^a^	0.263^a^	0.277^a^	0.158^a^	1	3.41	0.61

a . Correlation is significant at the 0.01 level (2-tailed).

The associations among the study's variables are presented in Table 4. According to Pallant [82], multicollinearity was ruled out as the variance inflation factors (VIF) ranged from 1.14 to 5.93, and the correlation between important variables was less than 0.80. The findings demonstrate the extent to which motivations for using instant messaging and digital socializing factors are linked among Ethiopian university students. Pearson correlation coefficients were utilized. The data revealed significant correlations between aspects such as enjoyment, affection, inclusion, escape, relaxation, and socializing, with a correlation coefficient of 0.01. Statistically significant correlations were found between pleasure and affection (r = 0.420∗∗), inclusion and escape (r = 0.431∗∗), escape and control (r = 0.162∗∗), escape and socialization (r = 0.263∗∗), relaxation and socialization (r = 0.277∗∗), and control and socialization (r = 0.384∗∗). Additionally, significant correlations were observed between pleasure and inclusion (r = 0.399∗∗), affection and escape (r = 0.379∗∗), affection and relaxation (r = 0.290∗∗), affection and control (r = 0.189∗∗), affection and socialization (r = 0.384∗∗), inclusion and escape (r = 0.358∗∗), inclusion and relaxation (r = 0.329∗∗), inclusion and socialization (r = 0.309∗∗), and pleasure and socialization (r = 0.384∗∗). However, pleasure and control (r = 0.082), inclusion and control (r = 0.104), and relaxation and control (r = 0.088) did not exhibit significant relationships at the 0.01 level. The results indicate that control is not significantly related to pleasure, inclusion, and relaxation of the ICM sub-scales.

Table 5 indicates the standard coefficients (Beta) of the direct relationship with the use of DS with ICM factors, ranging from 0.063 to 3.478. The constant result shows the factors that affect DS in using SNs among Arba Minch University students in Ethiopia. Each factor's results show pleasure (B = 0.239, t = 3.478, p = 0.001); affection (B = 0.111, t = 1.739, p = 0.083); inclusion (B = 0.141, t = 2.270, p = 0.024); escape (B = 0.017, t = 0.260, p = 0.795); relaxation (B = 0.063, t = 0.966, p = 0.335); and control (B = 0.095, t = 1.705, p = 0.089). Additionally, the collinearity statistics show a good fit of the direct relationship of the variables of ICM with the DS variable.

**Table 5:** Standard coefficients (Beta) of the direct relationship with the use of DS with ICM factors using Collinearity statistics (VIF).

Variables	Beta	T	Sig.	Collinearity statistics (VIF)
(Constant)		5.288	0.000	–
Pleasure	0.239	3.478	0.001	1.612
Affection	0.111	1.739	0.083	1.394
Inclusion	0.141	2.270	0.024	1.319
Escape	0.017	0.260	0.795	1.470
Relaxation	0.063	0.966	0.335	1.455
Control	0.095	1.705	0.089	1.049

Gender disparities in ICM and digital socialization among college students are evident in Table 6. Males had a mean enjoyment score of M = 3.26 (SD = 0.66), while females had a mean pleasure score of M = 3.28 (SD = 0.61). The mean affection score for males was M = 3.34 (SD = 0.64), compared to M = 3.42 (SD = 0.67) for females. Male inclusion scores averaged M = 3.20 (SD = 0.70), whereas female inclusion scores were M = 3.24 (SD = 0.61). On the escape scale, males scored M = 3.46 (SD = 0.67), while females scored M = 2.54 (SD = 0.73). Males had a mean relaxation score of M = 3.60 (SD = 0.71), while females had a mean relaxation score of M = 3.61 (SD = 0.61). The mean control score was M = 3.51 (SD = 0.75) for males and M = 3.64 (SD = 0.74) for females. The mean socialization score was M = 3.39 (SD = 0.61) for males and M = 3.47 (SD = 0.60) for females.

**Table 6:** Tests of Between-Subjects Effects (Df = 275) towards gender differences.

Variables	Gender	M	SD	MS	F	Sig.	B
Pleasure	Male	3.26	0.66	0.022	0.052	0.820	−0.020
	Female	3.28	0.61				
Affection	Male	3.34	0.64	0.353	0.837	0.361	−0.080
	Female	3.42	0.67				
Inclusion	Male	3.20	0.70	0.077	0.167	0.683	−0.037
	Female	3.24	0.61				
Escape	Male	3.46	0.67	0.365	0.775	0.379	−0.082
	Female	3.54	0.73				
Relaxation	Male	3.60	0.71	0.004	0.009	0.927	−0.009
	Female	3.61	0.61				
Control	Male	3.51	0.75	0.775	1.382	0.241	−0.119
	Female	3.64	0.74				
Socialization	Male	3.39	0.61	0.319	0.866	0.353	−0.076
	Female	3.47	0.60				

However, mean values alone cannot determine if there are statistically significant differences in socialization through DTs among Ethiopian university students and the mean values of ICM dimensions. To identify any significant variations in the mean scores regarding the reasons underlying IC and DS in SNs, a between-subjects effects analysis was conducted. Table 5 shows that there were no significant differences in the ICM dimensions at the following levels: inclusion = F(1, 275) = 0.167, p = 0.683; escape = F(1, 275) = 0.365, p = 0.775; relaxation = F(1, 275) = 0.009, p = 0.927; control = F(1, 275) = 1.382, p = 0.241; and socialization = F(1, 275) = 0.866, p = 0.353. Therefore, gender disparities did not have an impact on the reasons for IC and SNs in DTs.

### 4.2 FGD results

A thematic interpretation approach was utilized to assess the FGD data. The responses given by the participants in the focus group discussion were examined thematically using six questions provided by the researchers. Initially, the researchers familiarized themselves with the data by reading and re-reading it to gain a better understanding of the participants' responses and to structure the content for analysis. Next, the data was coded to allocate segments for analysis. Subsequently, the researchers identified, defined, and analyzed the themes present in the data, reviewing them for accuracy. Each theme was labeled to convey its meaning and significance. Finally, the findings were compiled and presented in Table 7 of the report.

**Table 7:** FGD responses using thematic descriptions.

FGD Questions	Respondents	Core theme(s)
How often do you use SNs?	- I always use my devices for about 2 h every day. - I use them occasionally, especially when I have assignments for my courses. - I rely on them to communicate with my family.	- Occasionally use
What are your primary reasons for using SNs?	- My primary reason is to entertain myself with my online friends. - I use it to search for academic information and do assignments. - I use it to explore new innovations and ideas written or uploaded by authors. - I use it to watch films and listen to music. - I use it to socialize with others. For example, I have many friends who live abroad.	- Reap the social benefits of communication (pleasure), - Desire to unwind (relaxation), - Fulfill needs for companionship (inclusion), -Exert power (control)
How do you feel SNs have impacted your ICM?	- I feel that SNs are important in daily life and necessary to connect with my friends and family. - I use SNs to communicate with my family, friends, and classmates. - I feel that SNs are a part of my academic journey. - I use it daily and I enjoy it.	- Basic need for socialization
How do you balance your time between SNs and face-to-face interactions?	- I always use my DTs during lunch and at night, while I communicate with my classmates during class times. - During rest time, I use my SNs to communicate with my family and friends.	- Used SNs in any circumstances
How do you perceive the role of SNs in building and maintaining relationships?	- If we use SNs for academic and business purposes, it is used to communicate with others. - It is very important to communicate with people across borders. - SN is a very useful technology, particularly for adolescents like me.	- Powerful to socialization development
Do you believe that SNs have made it easier or more difficult to establish genuine connections with others?	- Yes, I believe that SNs can present challenges if we are not using them for positive purposes. - The connection between people on SNs is easily formed when we engage in positive discussions and interactions. - SNs play a crucial role in establishing positive connections with others.	- It is conditional for use it

When asked “How often do you use SNs?” in response to the first question, many FGD participants indicated in Table 6 that they use SNs sometimes. One responder, for instance, said, “I use them occasionally, especially when I have assignments for my courses.” This demonstrates how university students' motivations for engaging in IC might occasionally be tied to homework and social concerns. The second question, “What are your primary reasons for using SNs?” was likewise addressed by the respondents. While some use them for relaxation, others use them for pleasure. When using SNs, some people also utilize them for inclusion and control purposes. Two respondents said, for instance, “I use it to watch films and listen to music” in response to this question. I have a lot of acquaintances who are expatriates, for instance. This indicates that engaging in interpersonal contact is a means for university students to amuse themselves and interact with others.

Furthermore, the researchers brought up problems about face-to-face socializing and the DS. A basic need for human sociability through various modes of communication in social media channels was cited by the present study's FGD participants in response to the third question, “How do you feel SNs have impacted your ICM?” As for the fourth question, “How do you balance your time between SNs and face-to-face interactions?” research participants who participated in the focus group discussion also responded, saying that SNs are great for socializing and communicating at any time. In response to the fifth question, “How do you perceive the role of SNs in building and maintaining relationships?” it was said that SNs have a significant impact on the formation and upkeep of socialization through their use. As an illustration, a few respondents said, “It is crucial to communicate with people across borders” and “If we use SNs for academic and business purposes, they are used to communicate with others.” This suggests that socializing and interpersonal communication can benefit from the usage of SNs. That depends on the circumstances of DT's socialization growth, was the response to the last question, “Do you believe that SNs have made it easier or more difficult to establish genuine connections with others?” As an instance, a participant expressed, “When we engage in positive discussions and interactions, the connection between people on SNs is easily formed."

In summary, the results suggest that student ICM and DS may have an impact on policy and reorient attention from academic challenges to students' digital psychological health and interpersonal communication practices inside Ethiopia's higher education system. Although these results might not hold true for all learners, they do provide insightful information that might be helpful in certain situations. Furthermore, among college students, the study demonstrates a relationship between DS and ICM. Findings from recent research show that while there is no substantial association between control and enjoyment, escape, or relaxation, there is one between ICM dimensions and DS. Also, the fact that many college students utilize SNs technology for leisure, socializing, and relaxation among other reasons, influenced the findings from the qualitative data.

## 5 Discussion

The first research question of the study was aimed to explore the impact of ICM on Ethiopian SNs in relation to DS behavior. The study hypothesized that a significant number of undergraduate students engage in DS due to ICM factors. Data was collected from Ethiopian university students through self-report questionnaires to test this hypothesis. Descriptive values indicated a moderate level (as outlined in Refs. [1,77] 1–2.39 = low; 2.40–3.69 = moderate; 3.70–5.00 = high) after data collection, and relevant statistical methods were used to evaluate the findings. The findings of this study on the prevalence of ICM and SNU with DS behavior among AMU students revealed that 30.00 %–32.85 % of respondents had high ICM, while 36.1 %–43.68 % had moderate ICM, and 24.91 %–33.21 % had low ICM. On average, more than half of the respondents had high and moderate ICM, including pleasure, affection, inclusion, relaxation, escape, and control.

The prevalence of ICM among adults was 42.53 %. The study by Man et al. [42] concluded that the prevalence of online social media usage was 37.43 %, with factors associated with DTs at 44.32 %. These results indicated that SNU in adults was influenced by DTs factors. However, the findings of this study are higher than those of a study conducted by Hardjati and Febrianita [11], which found that 32.23 % of teenagers experienced social media platforms, and the prevalence of SNs was 37.73 %. Weaverdyck and Parkinson [14] suggest that DTs have replaced traditional socialization processes in the 2000s, influencing social network relationships, behaviors, mental processes, and patterns. Similarly, DTs influence social network relationships, behaviors, mental processes, and behavioral patterns [16]. Interactions within digital technologies are still influenced by digital socialization. Therefore, the impact of ICM subscales on DS behavior was significantly positive at the 0.01 level.

Similarly, Diehl and Bose [7] highlight the lack of research on how these incentives affect SNs in mediated contexts, with most ICM studies focusing on face-to-face interactions and their prevalence and effectiveness in the digital realm. One exception is the rare examination [26] regarding DT-mediated communication. Considering these previous studies alongside the survey results, it becomes evident that ICM is a complex issue among high school students in Ethiopia and beyond.

The second research question of the study aimed to determine how ICM subscales affected Ethiopian university students' socialization in DTs. To gain a better understanding, the hypothesis was designed to investigate how ICM factors impact the engagement of undergraduate students in using DS. A statistically significant difference was found in the ICM subscales, except for the control subscale, based on the descriptive statistics from the self-reported questionnaire and the focus group discussion. The partial eta squared effect sizes of these differences ranged from minor to significant. The study's findings showed a positive relationship between ICM and SN-mediated socialization among Ethiopian college students. Except for control, which had no statistically significant relationship with pleasure, escape, or relaxation factors, every ICM—pleasure, affection, escape, relaxation, and inclusion—was strongly connected with one another. This strong and positive link indicates that the ICM aspects influence each other. Despite some limitations, university students generally have positive attitudes towards DS [20,62]. Students view ICMs as crucial as a second family when they attend colleges. In universities, students face numerous challenges, and trust and a sense of family are essential aspects that strong ICMs help students develop [58,68,71]. Through trust, students can build relationships with their classmates and DTs worldwide. Open communication plays a vital role in fostering trust among students at universities, as highlighted by Anyim [28] and Caballini et al. [39]. Additionally, the message in ICMs is the component transmitted from one individual to another [38]. A lack of technological resources, such as laptops and smartphones, as well as insufficient funds for phone credit recharges, posed challenges for instructors and university students navigating online learning [13].

The results align with previous research demonstrating a strong positive connection between ICM factors [9,10,20]. There is limited research on the link between ICM and socialization in SNs among Ethiopian university students, but the findings may coincide with studies exploring the motivations behind IC. For example, Wei et al. [17] suggest that being part of a DS influences members' thoughts, feelings, and actions, impacting community trust and collective efficacy. While members of DTs may share common interests, their socialization methods vary [9,11,12]. Pham et al. [21] found a significant correlation between the use of SNs and ICM. Additionally, Wang et al.'s [18] research supports the substantial relationship between ICM levels and online SNs. DTs are emerging as crucial socialization agents that compete with others and influence attitudes and behaviors, particularly among adult college and university students, as noted by Weaverdyck and Parkinson [14]. According to the social learning theory [18], a community is a social organization where activity occurs through ICM. This theory also suggests that SNs is vital for learning participation, with communities of practice impacting knowledge and cognitive development [19].

Finally, the third research question addressed the gender differences in undergraduate students' ICM using DTs. The findings demonstrated a positive response to the hypothesized issue. Since the introduction of ICM, several studies have further supported its factor structure and its associations with variables such as age [3,11], communication apprehension [4], loneliness [69], contextual age [8,70], locus of control, and general communication [10,70]. However, the findings indicated no statistically significant correlations between gender-related ICM and socialization. Therefore, gender has minimal impact on ICM or socialization through Social Networks (SNs) among university students in Ethiopia. SNs play a crucial role in helping college students navigate DTs and effectively engage with information, as suggested by Alexopoulos et al. [4]; this conclusion is consistent with previous research. In conclusion, university students should regularly assess the impact of ICM on socialization through DTs and make necessary adjustments [6]. Gender disparities among participating students do not indicate significant differences in ICM and socialization through DTs.

## 6 Limitations and future research

Some recommendations for further research are made in light of the study's shortcomings. The primary limitation of the study was its exploration of ICM's approach to socialization in SNs, which may not fully represent all university students or their socialization experiences in an academic setting. The representativeness of the sample and the generalizability of the findings are two further constraints that must be acknowledged and taken into account. Self-selection sample bias may be present because the data were gathered using a self-reported questionnaire. Furthermore, because the participants were ICM, there may have been an engaged and professional bias among them, leading to an imbalance in the sample concerning the study's ecological location. To fill this knowledge vacuum and shed more light on the possible function that DTs could have in academic interaction, future research should be conducted using homogenous participants from language teachers and students.

Furthermore, this study has limitations that need to be addressed in further research. Firstly, because the study exclusively focused on Ethiopian university students, it may not provide a comprehensive understanding of ICM and socialization in SNs regarding gender disparities. Additionally, the results may not be applicable to other populations, and the sample used in this study may not be typical. The use of questionnaires and focus group discussions (FGD) in data collection could introduce self-selection sample bias. The study's location may have also influenced the findings. To gain a more comprehensive understanding of the potential role of SNs in DS, future research endeavors should include a wider range of subjects, from elementary to upper secondary school pupils. It would be beneficial for future scholars to examine university students in more detail, not limited to those at Arba Minch University in Ethiopia. The results of this study could also be relevant to other developing nations grappling with DTs associated with DS in relation to ICM. The study suggests that ICM should be taught to university students to promote the development of their digital resilience and self-efficacy.

## 7 Conclusion

Data was gathered from undergraduate students enrolled at Arba Minch University using a self-report questionnaire as part of the study's investigation of digital storytelling (DS) through integrated content management (ICM) in SNs. The data revealed a strong association between ICM characteristics and socialization, indicating that integrated technology is essential for effective DS and socializing in Ethiopian university settings. Therefore, the relationship between DS and ICM can be influenced by the usefulness and accessibility of digital technologies (DTs). Students are more likely to accept these technologies if they are user-friendly, easily accessible, and align with their ICM. Additionally, students will find it easier to incorporate these tools into their socializing process if they are readily available and compatible with the existing digital infrastructure. Consequently, students are encouraged to incorporate DTs into their coursework and leverage their interactions with preferred DS and ICM for their benefit.

Several suggestions can be implemented to enhance ICM with socializing. Firstly, university authorities recommend that DS include DTs that combine technical expertise with educational methodologies. Workshops, conferences, and online courses tailored to the diverse needs and interests of instructors can facilitate this goal. Secondly, schools should invest in user-friendly and intuitive DTs. Instructors should be involved in the decision-making process to ensure that these tools support their educational objectives. Moreover, educational institutions must ensure that these resources are easily accessible and compatible with the existing technological framework. It is essential for districts to ensure equitable access to technology and internet connectivity for all students, and DTs should be provided to help instructors become proficient in ICM. Other researchers interested in DS and ICM in SNs may also find the study's findings beneficial.

## CRediT authorship contribution statement

**Hailay Tesfay Gebremariam:** Writing – review & editing, Writing – original draft, Visualization, Validation, Project administration, Investigation, Formal analysis, Conceptualization. **Paulos Dea:** Writing – review & editing, Supervision, Project administration, Investigation, Data curation. **Metasebya Gonta:** Writing – review & editing, Supervision, Methodology, Data curation.

## Declarations

### Ethics approval and consent to participate

Human participants involved in the studies were informed that they could voluntarily take part in the study, and that the results would be used for educational research purposes. The patients/participants provided their written informed consent to participate in this study. The study's ethical progress was also reviewed by two senior professors and approved by Department Graduate Committee (Ref. No.: PSD/251/16) of Psychology Department at Wolaita Sodo University.

## Conflict of competing interests

The authors have declared that they have no potential conflicts of interest with regards to the research, authorship, and/or publication of this article.

## Data availability and materials

All data are available upon request from the editor, and the corresponding author can provide them.

## Funding

No funding was received during the conduct of this study.

## Declaration of competing interest

The authors declare that they have no known competing financial interests or personal relationships that could have appeared to influence the work reported in this paper.
